# Diethyl {[5-(2,4-dichloro­phen­yl)-1,3,4-thia­diazol-2-ylamino](4-methoxy­phenyl)methyl}­phospho­nate

**DOI:** 10.1107/S1600536809012471

**Published:** 2009-04-08

**Authors:** Yao Wang, Rong Wan, Li-He Yin, Feng Han, Peng Wang

**Affiliations:** a Department of Applied Chemistry, College of Science, Nanjing University of Technology, No.5 Xinmofan Road, Nanjing, Nanjing 210009, People’s Republic of China

## Abstract

The title compound, C_20_H_22_Cl_2_N_3_O_4_PS, was synthesized by the reaction of *N*-(4-methoxy­benzyl­idene)-5-(2,4-dichloro­phenyl)-1,3,4-thia­diazol-2-amine and diethyl phosphite. In the crystal, inter­molecular C—H⋯O and N—H⋯O hydrogen bonds link the mol­ecules.

## Related literature

For applications of thia­diazole ligands, see: Nakagawa *et al.* (1996 ▶); Omar *et al.* (1986 ▶); Sato *et al.* (1991 ▶); Wang *et al.* (1999 ▶). For related structures, see: Wan *et al.* (2007 ▶); Yin *et al.* (2008 ▶). For bond-length data, see: Allen *et al.* (1987 ▶).
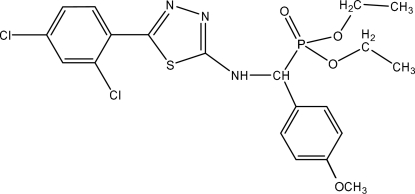


## Experimental

### 

#### Crystal data

C_20_H_22_Cl_2_N_3_O_4_PS
*M* *_r_* = 502.34
Triclinic, 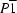

*a* = 9.7100 (19) Å
*b* = 11.825 (2) Å
*c* = 11.845 (2) Å
α = 98.74 (3)°
β = 112.16 (3)°
γ = 103.05 (3)°
*V* = 1183.9 (4) Å^3^
*Z* = 2
Mo *K*α radiation
μ = 0.46 mm^−1^
*T* = 293 K
0.30 × 0.20 × 0.10 mm

#### Data collection

Enraf–Nonius CAD-4 diffractometer
Absorption correction: ψ scan (North *et al.*, 1968 ▶) *T*_min_ = 0.874, *T*_max_ = 0.955
4592 measured reflections
4316 independent reflections
2864 reflections with *I* > 2σ(*I*)
*R*_int_ = 0.054
3 standard reflections every 200 reflections intensity decay: 1%

#### Refinement

*R*[*F* ^2^ > 2σ(*F* ^2^)] = 0.069
*wR*(*F* ^2^) = 0.157
*S* = 1.02
4316 reflections
277 parameters
H-atom parameters constrained
Δρ_max_ = 0.56 e Å^−3^
Δρ_min_ = −0.98 e Å^−3^

### 

Data collection: *CAD-4 EXPRESS* (Enraf–Nonius, 1989 ▶); cell refinement: *CAD-4 EXPRESS*; data reduction: *XCAD4* (Harms & Wocadlo, 1995 ▶); program(s) used to solve structure: *SHELXS97* (Sheldrick, 2008 ▶); program(s) used to refine structure: *SHELXL97* (Sheldrick, 2008 ▶); molecular graphics: *SHELXTL* (Sheldrick, 2008 ▶); software used to prepare material for publication: *SHELXL97*.

## Supplementary Material

Crystal structure: contains datablocks _global, I. DOI: 10.1107/S1600536809012471/ez2165sup1.cif

Structure factors: contains datablocks I. DOI: 10.1107/S1600536809012471/ez2165Isup2.hkl

Additional supplementary materials:  crystallographic information; 3D view; checkCIF report

## Figures and Tables

**Table 1:** Hydrogen-bond geometry (Å, °)

*D*—H⋯*A*	*D*—H	H⋯*A*	*D*⋯*A*	*D*—H⋯*A*
N1—H1*A*⋯O3^i^	0.86	2.00	2.805 (5)	156
C10—H10*A*⋯O4^ii^	0.93	2.53	3.431 (7)	163

Symmetry codes: (i) 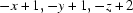; (ii) 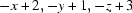.

**Fig. 1.**
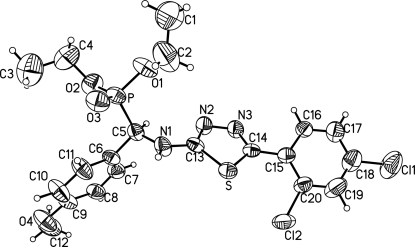
A view of the molecular structure of (I). Displacement ellipsoids are drawn at the 50% probability level.

**Fig. 2.**
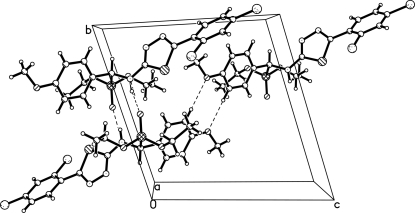
Partial packing view showing the hydrogen-bonded network. Dashed lines indicate intermolecular C—H···O and N—H···O hydrogen bonds.

## supplementary crystallographic information

### Comment

1,3,4-Thiadiazole derivatives represent an interesting class of compounds possessing broad spectrum biological activities (Nakagawa *et al.*, 1996). These compounds are known to exhibit diverse biological effects, such as insecticidal and fungicidal activities (Wang *et al.*, 1999). They can also be widely used in the field of medicine (Sato *et al.*, 1991), such as for anti-cancer drugs (Omar *et al.*, 1986).

We report here the crystal structure of the title compound,(I). The molecular structure of (I) is shown in Fig.1. Bond lengths are in the normal ranges (Allen *et al.*,  1987). The dihedral angle between the C15—C20 and S/C13/N2/N3/C14 is 32.4 (3)°, which shows that these two aromatic rings are not in the same plane. This dihedral angle is bigger than other phosphonate compounds, which is 7.54 (3)° (Wan *et al.*, 2007) and 5.3 (2)° (Yin *et al.*, 2008). There are intermolecular C—H···O and N—H···O hydrogen bonds (Fig. 2), which form chains along the *b* axis in the crystal.

### Experimental

*N*-(4–methoxyphenyl)-5-(2,4-dichlorophenyl)-1,3,4-thiadiazol-2-amine (2 mmol) and diethyl phosphite (5 mmol) were mixed in a 25 ml flask, and kept in an oil bath at 90°C for 6 h. After cooling, the crude product (I) precipitated and was filtered. Pure compound (I) was obtained by crystallization from ethanol (20 ml). Crystals of (I) suitable for X-ray diffraction were obtained by slow evaporation of an acetone solution.

### Refinement

All H atoms bonded to the C atoms were placed geometrically at the distances of 0.93–0.97 Å and included in the refinement in riding motion approximation with *U*_iso_(H) = 1.2 or 1.5*U*_eq_ of the carrier atom.

### Crystal data

**Table:**

C_20_H_22_Cl_2_N_3_O_4_PS	*F*(000) = 520
*M**_r_* = 502.34	*D*_x_ = 1.409 Mg m^−^^3^
Triclinic, *P*1	Melting point: 59365 K
*a* = 9.7100 (19) Å	Mo *K*α radiation, λ = 0.71073 Å
*b* = 11.825 (2) Å	Cell parameters from 25 reflections
*c* = 11.845 (2) Å	θ = 9–13°
α = 98.74 (3)°	µ = 0.46 mm^−^^1^
β = 112.16 (3)°	*T* = 293 K
γ = 103.05 (3)°	Block, colorless
*V* = 1183.9 (4) Å^3^	0.30 × 0.20 × 0.10 mm
*Z* = 2	

### Data collection

**Table:**

Enraf–Nonius CAD-4 diffractometer	2864 reflections with *I* > 2σ(*I*)
Radiation source: fine-focus sealed tube	*R*_int_ = 0.054
graphite	θ_max_ = 25.3°, θ_min_ = 1.8°
ω/2θ scans	*h* = 0→11
Absorption correction: ψ scan (North *et al.*, 1968)	*k* = −14→13
*T*_min_ = 0.874, *T*_max_ = 0.955	*l* = −14→13
4592 measured reflections	3 standard reflections every 200 reflections
4316 independent reflections	intensity decay: 1%

### Refinement

**Table:**

Refinement on *F*^2^	Primary atom site location: structure-invariant direct methods
Least-squares matrix: full	Secondary atom site location: difference Fourier map
*R*[*F*^2^ > 2σ(*F*^2^)] = 0.069	Hydrogen site location: inferred from neighbouring sites
*wR*(*F*^2^) = 0.157	H-atom parameters constrained
*S* = 1.02	*w* = 1/[σ^2^(*F*_o_^2^) + (0.0281*P*)^2^ + 3.5816*P*] where *P* = (*F*_o_^2^ + 2*F*_c_^2^)/3
4316 reflections	(Δ/σ)_max_ < 0.001
277 parameters	Δρ_max_ = 0.56 e Å^−^^3^
0 restraints	Δρ_min_ = −0.97 e Å^−^^3^

### Special details

**Table:**

Geometry. All e.s.d.'s (except the e.s.d. in the dihedral angle between two l.s. planes) are estimated using the full covariance matrix. The cell e.s.d.'s are taken into account individually in the estimation of e.s.d.'s in distances, angles and torsion angles; correlations between e.s.d.'s in cell parameters are only used when they are defined by crystal symmetry. An approximate (isotropic) treatment of cell e.s.d.'s is used for estimating e.s.d.'s involving l.s. planes.
Refinement. Refinement of *F*^2^ against ALL reflections. The weighted *R*-factor *wR* and goodness of fit *S* are based on *F*^2^, conventional *R*-factors *R* are based on *F*, with *F* set to zero for negative *F*^2^. The threshold expression of *F*^2^ > σ(*F*^2^) is used only for calculating *R*-factors(gt) *etc*. and is not relevant to the choice of reflections for refinement. *R*-factors based on *F*^2^ are statistically about twice as large as those based on *F*, and *R*- factors based on ALL data will be even larger.

### Fractional atomic coordinates and isotropic or equivalent isotropic displacement parameters (Å^2^)

**Table:**

	*x*	*y*	*z*	*U*_iso_*/*U*_eq_	
Cl1	0.1915 (3)	−0.0588 (2)	0.04559 (17)	0.1292 (9)	
Cl2	0.60795 (15)	0.19354 (15)	0.51042 (15)	0.0812 (5)	
S	0.45871 (13)	0.26753 (10)	0.68952 (11)	0.0518 (3)	
P	0.28539 (13)	0.35163 (11)	1.04983 (12)	0.0508 (3)	
N1	0.4404 (4)	0.3110 (3)	0.9121 (3)	0.0524 (10)	
H1A	0.4934	0.3843	0.9247	0.063*	
O1	0.1252 (4)	0.2999 (3)	0.9312 (4)	0.0754 (11)	
C1	−0.0855 (9)	0.3620 (8)	0.8045 (8)	0.112	
H1B	−0.1550	0.2881	0.7427	0.168*	
H1C	−0.1008	0.4286	0.7694	0.168*	
H1D	−0.1069	0.3689	0.8777	0.168*	
N2	0.2791 (5)	0.1297 (3)	0.7581 (3)	0.0541 (10)	
O2	0.2564 (4)	0.2931 (3)	1.1510 (3)	0.0698 (10)	
C2	0.0749 (9)	0.3624 (9)	0.8405 (8)	0.123 (3)	
H2A	0.0851	0.3269	0.7658	0.148*	
H2B	0.1427	0.4451	0.8733	0.148*	
O3	0.3460 (4)	0.4837 (3)	1.0857 (3)	0.0621 (9)	
N3	0.2468 (4)	0.0703 (3)	0.6370 (3)	0.0541 (10)	
C3	0.2866 (11)	0.4070 (7)	1.3512 (7)	0.127 (3)	
H3A	0.3578	0.3667	1.3943	0.191*	
H3B	0.2285	0.4241	1.3981	0.191*	
H3C	0.3443	0.4808	1.3440	0.191*	
O4	0.9613 (4)	0.3317 (3)	1.4496 (4)	0.0826 (12)	
C4	0.1777 (9)	0.3289 (7)	1.2240 (7)	0.100 (2)	
H4A	0.1134	0.2577	1.2320	0.120*	
H4B	0.1091	0.3715	1.1796	0.120*	
C5	0.4108 (5)	0.2749 (4)	1.0145 (4)	0.0461 (10)	
H5A	0.3525	0.1888	0.9834	0.055*	
C6	0.5616 (5)	0.2897 (4)	1.1290 (4)	0.0454 (10)	
C7	0.6196 (5)	0.1950 (4)	1.1435 (5)	0.0572 (12)	
H7A	0.5671	0.1221	1.0821	0.069*	
C8	0.7548 (5)	0.2052 (4)	1.2475 (5)	0.0583 (12)	
H8A	0.7942	0.1406	1.2546	0.070*	
C9	0.8308 (5)	0.3130 (4)	1.3412 (5)	0.0569 (12)	
C10	0.7780 (6)	0.4085 (4)	1.3276 (5)	0.0735 (16)	
H10A	0.8300	0.4811	1.3896	0.088*	
C11	0.6447 (6)	0.3977 (4)	1.2197 (5)	0.0670 (15)	
H11A	0.6114	0.4647	1.2088	0.080*	
C13	0.3876 (5)	0.2336 (4)	0.7974 (4)	0.0441 (10)	
C14	0.3304 (5)	0.1296 (4)	0.5888 (4)	0.0418 (9)	
C15	0.3017 (5)	0.0854 (4)	0.4561 (4)	0.0468 (10)	
C16	0.1499 (5)	0.0152 (4)	0.3671 (4)	0.0515 (11)	
H16A	0.0708	−0.0020	0.3939	0.062*	
C17	0.1154 (7)	−0.0281 (5)	0.2443 (5)	0.0700 (15)	
H17A	0.0133	−0.0731	0.1882	0.084*	
C18	0.2304 (8)	−0.0064 (5)	0.2005 (5)	0.0770 (17)	
C19	0.3822 (8)	0.0635 (6)	0.2868 (6)	0.0856 (19)	
H19A	0.4608	0.0802	0.2595	0.103*	
C20	0.4161 (6)	0.1077 (4)	0.4115 (5)	0.0553 (12)	
C12	1.0077 (7)	0.2351 (6)	1.4818 (7)	0.089 (2)	
H12A	0.9175	0.1691	1.4638	0.133*	
H12B	1.0760	0.2571	1.5703	0.133*	
H12C	1.0619	0.2116	1.4339	0.133*	

### Atomic displacement parameters (Å^2^)

**Table:**

	*U*^11^	*U*^22^	*U*^33^	*U*^12^	*U*^13^	*U*^23^
Cl1	0.1449 (18)	0.1336 (17)	0.0748 (11)	−0.0205 (14)	0.0628 (12)	−0.0134 (10)
Cl2	0.0454 (7)	0.0980 (11)	0.0881 (10)	0.0027 (7)	0.0353 (7)	0.0024 (8)
S	0.0407 (6)	0.0465 (6)	0.0540 (6)	−0.0047 (5)	0.0178 (5)	0.0081 (5)
P	0.0381 (6)	0.0437 (6)	0.0588 (7)	0.0023 (5)	0.0147 (5)	0.0120 (5)
N1	0.052 (2)	0.0392 (19)	0.045 (2)	−0.0059 (16)	0.0124 (17)	0.0063 (16)
O1	0.0436 (19)	0.071 (2)	0.083 (3)	0.0092 (17)	0.0032 (18)	0.017 (2)
C1	0.112	0.112	0.112	0.035	0.048	0.029
N2	0.058 (2)	0.043 (2)	0.049 (2)	−0.0044 (17)	0.0235 (18)	0.0071 (16)
O2	0.076 (2)	0.061 (2)	0.080 (2)	0.0123 (19)	0.045 (2)	0.0251 (19)
C2	0.108 (6)	0.143 (7)	0.086 (5)	0.030 (6)	0.012 (4)	0.030 (5)
O3	0.055 (2)	0.0462 (18)	0.077 (2)	0.0073 (15)	0.0238 (17)	0.0175 (16)
N3	0.052 (2)	0.046 (2)	0.049 (2)	−0.0017 (17)	0.0170 (18)	0.0078 (17)
C3	0.167 (9)	0.098 (6)	0.123 (7)	0.036 (6)	0.073 (7)	0.027 (5)
O4	0.060 (2)	0.057 (2)	0.091 (3)	0.0136 (18)	−0.007 (2)	0.019 (2)
C4	0.112 (6)	0.104 (5)	0.117 (6)	0.041 (5)	0.076 (5)	0.037 (5)
C5	0.045 (2)	0.037 (2)	0.046 (2)	−0.0004 (18)	0.0152 (19)	0.0106 (18)
C6	0.035 (2)	0.040 (2)	0.053 (2)	0.0051 (18)	0.0130 (19)	0.0170 (19)
C7	0.049 (3)	0.046 (3)	0.068 (3)	0.010 (2)	0.023 (2)	0.005 (2)
C8	0.042 (3)	0.055 (3)	0.073 (3)	0.019 (2)	0.017 (2)	0.017 (2)
C9	0.037 (2)	0.047 (3)	0.071 (3)	0.008 (2)	0.010 (2)	0.015 (2)
C10	0.060 (3)	0.042 (3)	0.077 (4)	0.009 (2)	−0.006 (3)	0.005 (2)
C11	0.058 (3)	0.042 (3)	0.069 (3)	0.015 (2)	−0.003 (2)	0.005 (2)
C13	0.031 (2)	0.041 (2)	0.049 (2)	0.0038 (17)	0.0097 (18)	0.0123 (18)
C14	0.032 (2)	0.038 (2)	0.052 (2)	0.0068 (17)	0.0179 (18)	0.0104 (18)
C15	0.044 (2)	0.039 (2)	0.055 (2)	0.0091 (18)	0.021 (2)	0.0109 (19)
C16	0.045 (2)	0.046 (2)	0.051 (2)	0.001 (2)	0.018 (2)	0.003 (2)
C17	0.072 (4)	0.055 (3)	0.059 (3)	−0.008 (3)	0.022 (3)	0.005 (2)
C18	0.091 (4)	0.062 (3)	0.071 (3)	0.001 (3)	0.044 (3)	0.008 (3)
C19	0.087 (4)	0.081 (4)	0.091 (4)	0.002 (3)	0.060 (4)	0.006 (3)
C20	0.051 (3)	0.052 (3)	0.063 (3)	0.006 (2)	0.032 (2)	0.008 (2)
C12	0.063 (4)	0.073 (4)	0.110 (5)	0.027 (3)	0.007 (3)	0.037 (4)

### Geometric parameters (Å, °)

**Table:**

Cl1—C18	1.709 (6)	C4—H4A	0.9700
Cl2—C20	1.736 (5)	C4—H4B	0.9700
S—C13	1.723 (4)	C5—C6	1.526 (6)
S—C14	1.733 (4)	C5—H5A	0.9800
P—O3	1.469 (3)	C6—C7	1.368 (6)
P—O2	1.553 (4)	C6—C11	1.378 (6)
P—O1	1.559 (4)	C7—C8	1.385 (7)
P—C5	1.803 (5)	C7—H7A	0.9300
N1—C13	1.354 (5)	C8—C9	1.388 (7)
N1—C5	1.449 (5)	C8—H8A	0.9300
N1—H1A	0.8600	C9—C10	1.348 (7)
O1—C2	1.395 (9)	C10—C11	1.398 (7)
C1—C2	1.450 (8)	C10—H10A	0.9300
C1—H1B	0.9600	C11—H11A	0.9300
C1—H1C	0.9600	C14—C15	1.475 (6)
C1—H1D	0.9600	C15—C20	1.391 (6)
N2—C13	1.306 (5)	C15—C16	1.408 (6)
N2—N3	1.380 (5)	C16—C17	1.349 (7)
O2—C4	1.430 (7)	C16—H16A	0.9300
C2—H2A	0.9700	C17—C18	1.388 (8)
C2—H2B	0.9700	C17—H17A	0.9300
N3—C14	1.301 (5)	C18—C19	1.399 (8)
C3—C4	1.475 (7)	C19—C20	1.373 (7)
C3—H3A	0.9600	C19—H19A	0.9300
C3—H3B	0.9600	C12—H12A	0.9600
C3—H3C	0.9600	C12—H12B	0.9600
O4—C9	1.365 (6)	C12—H12C	0.9600
O4—C12	1.374 (7)		
			
C13—S—C14	87.0 (2)	C11—C6—C5	121.9 (4)
O3—P—O2	116.2 (2)	C6—C7—C8	121.6 (5)
O3—P—O1	113.2 (2)	C6—C7—H7A	119.2
O2—P—O1	104.1 (2)	C8—C7—H7A	119.2
O3—P—C5	115.3 (2)	C7—C8—C9	119.3 (5)
O2—P—C5	101.5 (2)	C7—C8—H8A	120.4
O1—P—C5	105.2 (2)	C9—C8—H8A	120.4
C13—N1—C5	122.3 (3)	C10—C9—O4	115.6 (4)
C13—N1—H1A	118.8	C10—C9—C8	120.2 (5)
C5—N1—H1A	118.8	O4—C9—C8	124.2 (4)
C2—O1—P	122.8 (4)	C9—C10—C11	119.6 (5)
C2—C1—H1B	109.5	C9—C10—H10A	120.2
C2—C1—H1C	109.5	C11—C10—H10A	120.2
H1B—C1—H1C	109.5	C6—C11—C10	121.4 (5)
C2—C1—H1D	109.5	C6—C11—H11A	119.3
H1B—C1—H1D	109.5	C10—C11—H11A	119.3
H1C—C1—H1D	109.5	N2—C13—N1	124.0 (4)
C13—N2—N3	111.7 (4)	N2—C13—S	114.6 (3)
C4—O2—P	126.3 (4)	N1—C13—S	121.4 (3)
O1—C2—C1	113.6 (7)	N3—C14—C15	121.0 (4)
O1—C2—H2A	108.8	N3—C14—S	113.4 (3)
C1—C2—H2A	108.8	C15—C14—S	125.3 (3)
O1—C2—H2B	108.8	C20—C15—C16	116.7 (4)
C1—C2—H2B	108.8	C20—C15—C14	124.2 (4)
H2A—C2—H2B	107.7	C16—C15—C14	119.1 (4)
C14—N3—N2	113.4 (3)	C17—C16—C15	122.2 (5)
C4—C3—H3A	109.5	C17—C16—H16A	118.9
C4—C3—H3B	109.5	C15—C16—H16A	118.9
H3A—C3—H3B	109.5	C16—C17—C18	120.8 (5)
C4—C3—H3C	109.5	C16—C17—H17A	119.6
H3A—C3—H3C	109.5	C18—C17—H17A	119.6
H3B—C3—H3C	109.5	C17—C18—C19	118.2 (5)
C9—O4—C12	119.5 (4)	C17—C18—Cl1	122.4 (5)
O2—C4—C3	112.8 (6)	C19—C18—Cl1	119.5 (4)
O2—C4—H4A	109.0	C20—C19—C18	120.6 (5)
C3—C4—H4A	109.0	C20—C19—H19A	119.7
O2—C4—H4B	109.0	C18—C19—H19A	119.7
C3—C4—H4B	109.0	C19—C20—C15	121.5 (5)
H4A—C4—H4B	107.8	C19—C20—Cl2	116.8 (4)
N1—C5—C6	112.2 (3)	C15—C20—Cl2	121.7 (4)
N1—C5—P	109.5 (3)	O4—C12—H12A	109.5
C6—C5—P	113.9 (3)	O4—C12—H12B	109.5
N1—C5—H5A	106.9	H12A—C12—H12B	109.5
C6—C5—H5A	106.9	O4—C12—H12C	109.5
P—C5—H5A	106.9	H12A—C12—H12C	109.5
C7—C6—C11	117.8 (4)	H12B—C12—H12C	109.5
C7—C6—C5	120.3 (4)		
			
O3—P—O1—C2	24.0 (6)	C7—C6—C11—C10	−4.4 (8)
O2—P—O1—C2	151.1 (6)	C5—C6—C11—C10	176.5 (5)
C5—P—O1—C2	−102.7 (6)	C9—C10—C11—C6	2.9 (10)
O3—P—O2—C4	46.8 (6)	N3—N2—C13—N1	179.2 (4)
O1—P—O2—C4	−78.3 (5)	N3—N2—C13—S	0.2 (5)
C5—P—O2—C4	172.6 (5)	C5—N1—C13—N2	15.7 (7)
P—O1—C2—C1	−133.6 (6)	C5—N1—C13—S	−165.4 (3)
C13—N2—N3—C14	0.1 (6)	C14—S—C13—N2	−0.3 (4)
P—O2—C4—C3	−98.2 (7)	C14—S—C13—N1	−179.3 (4)
C13—N1—C5—C6	118.7 (4)	N2—N3—C14—C15	−174.2 (4)
C13—N1—C5—P	−113.8 (4)	N2—N3—C14—S	−0.4 (5)
O3—P—C5—N1	−58.4 (3)	C13—S—C14—N3	0.4 (4)
O2—P—C5—N1	175.2 (3)	C13—S—C14—C15	173.9 (4)
O1—P—C5—N1	67.0 (3)	N3—C14—C15—C20	−150.9 (5)
O3—P—C5—C6	68.1 (3)	S—C14—C15—C20	36.0 (7)
O2—P—C5—C6	−58.3 (3)	N3—C14—C15—C16	28.6 (7)
O1—P—C5—C6	−166.5 (3)	S—C14—C15—C16	−144.4 (4)
N1—C5—C6—C7	−93.9 (5)	C20—C15—C16—C17	−0.5 (7)
P—C5—C6—C7	141.0 (4)	C14—C15—C16—C17	180.0 (5)
N1—C5—C6—C11	85.2 (6)	C15—C16—C17—C18	1.0 (9)
P—C5—C6—C11	−39.9 (6)	C16—C17—C18—C19	−1.2 (9)
C11—C6—C7—C8	1.9 (7)	C16—C17—C18—Cl1	−179.6 (5)
C5—C6—C7—C8	−178.9 (4)	C17—C18—C19—C20	0.9 (10)
C6—C7—C8—C9	1.9 (8)	Cl1—C18—C19—C20	179.3 (5)
C12—O4—C9—C10	168.9 (6)	C18—C19—C20—C15	−0.4 (10)
C12—O4—C9—C8	−12.1 (9)	C18—C19—C20—Cl2	−179.9 (5)
C7—C8—C9—C10	−3.5 (8)	C16—C15—C20—C19	0.2 (8)
C7—C8—C9—O4	177.5 (5)	C14—C15—C20—C19	179.7 (5)
O4—C9—C10—C11	−179.8 (5)	C16—C15—C20—Cl2	179.6 (4)
C8—C9—C10—C11	1.1 (9)	C14—C15—C20—Cl2	−0.8 (7)

### Hydrogen-bond geometry (Å, °)

**Table:**

*D*—H···*A*	*D*—H	H···*A*	*D*···*A*	*D*—H···*A*
N1—H1A···O3^i^	0.86	2.00	2.805 (5)	156
C10—H10A···O4^ii^	0.93	2.53	3.431 (7)	163

Symmetry codes: (i) −*x*+1, −*y*+1, −*z*+2; (ii) −*x*+2, −*y*+1, −*z*+3.
